# Assessing Non-Specific Neck Pain through Pose Estimation from Images Based on Ensemble Learning

**DOI:** 10.3390/life13122292

**Published:** 2023-11-30

**Authors:** Jiunn-Horng Kang, En-Han Hsieh, Cheng-Yang Lee, Yi-Ming Sun, Tzong-Yi Lee, Justin Bo-Kai Hsu, Tzu-Hao Chang

**Affiliations:** 1 Department of Physical Medicine and Rehabilitation, Taipei Medical University Hospital, Taipei 110, Taiwan; jhk@tmu.edu.tw; 2 Graduate Institute of Nanomedicine and Medical Engineering, Taipei Medical University, Taipei 110, Taiwan; 3 Graduate Institute of Biomedical Informatics, Taipei Medical University, Taipei 110, Taiwan; 4 PlexBio Co., Ltd., Taipei 114, Taiwan; 5 Institute of Bioinformatics and Systems Biology, National Yang Ming Chiao Tung University, Hsinchu 300, Taiwan; 6 Department of Computer Science and Engineering, Yuan Ze University, Taoyuan 320, Taiwan; 7 Clinical Big Data Research Center, Taipei Medical University Hospital, Taipei 110, Taiwan

**Keywords:** non-specific neck pain, single camera, video recording, image analysis, pose estimation, machine learning, ensemble learning

## Abstract

Background: Mobile phones, laptops, and computers have become an indispensable part of our lives in recent years. Workers may have an incorrect posture when using a computer for a prolonged period of time. Using these products with an incorrect posture can lead to neck pain. However, there are limited data on postures in real-life situations. Methods: In this study, we used a common camera to record images of subjects carrying out three different tasks (a typing task, a gaming task, and a video-watching task) on a computer. Different artificial intelligence (AI)-based pose estimation approaches were applied to analyze the head’s yaw, pitch, and roll and coordinate information of the eyes, nose, neck, and shoulders in the images. We used machine learning models such as random forest, XGBoost, logistic regression, and ensemble learning to build a model to predict whether a subject had neck pain by analyzing their posture when using the computer. Results: After feature selection and adjustment of the predictive models, nested cross-validation was applied to evaluate the models and fine-tune the hyperparameters. Finally, the ensemble learning approach was utilized to construct a model via bagging, which achieved a performance with 87% accuracy, 92% precision, 80.3% recall, 95.5% specificity, and an AUROC of 0.878. Conclusions: We developed a predictive model for the identification of non-specific neck pain using 2D video images without the need for costly devices, advanced environment settings, or extra sensors. This method could provide an effective way for clinically evaluating poor posture during real-world computer usage scenarios.

## 1. Introduction

Neck pain is one of the most common musculoskeletal disorders, with an estimated prevalence rate of about 27.0 per 1000 population in 2019 [1]. Neck pain is a multifactorial disease. A lack of physical activity, long duration of computer usage, and psychological stress are recognized as risk factors for chronic neck pain [2]. Neck pain has become an increasing problem in North America, Western Europe, and East Asia in recent years [1]. Due to the coronavirus disease 2019 (COVID-19) pandemic in recent years, teleworking has become an emerging working mode that may aggravate this condition. Workers may assume incorrect or poor postures (asymmetrical posture, forward head, etc.) when using a computer. In addition, environmental and ergonomic factors such as the depth of the table, the height of the table, and the height of the chair play crucial roles in poor posture. Many studies focused on associations among neck pain, poor posture, spinal mobility, and exercise [3,4,5,6,7], and some methods of analyzing posture have been implemented utilizing advanced technology, such as depth cameras [8] or radar systems [9]. Although medical treatments and physiotherapy can effectively relieve neck pain, prevention through adequate patient education is essential for managing neck pain. Promoting self-awareness of poor posture in real-world settings could help patients correct these problems.

Many approaches have been explored to track and evaluate human posture, such as recording a subject’s posture with wearable devices [10,11,12]. These systems can assess a subject’s posture and record the subject’s abnormal movement patterns in clinical situations [13,14,15]. Image solutions that focus on non-contact posture tracking and evaluation without using wearable devices have also been adopted [16]. Abobakr et al. used video recordings to evaluate the working posture of workers [17]. Gao et al. used three-dimensional (3D) postural images to analyze the location and degree of sports injuries [18]. However, these approaches require special environments and equipment to collect data when applied in the real world, which could be costly.

De la Torre et al. published information captured by wearable sensors and used the data from 151 patients with 302 samples and 28 features to train a model to predict whether a subject has neck pain [10]. Chen et al. constructed a model to assess the sitting posture of students in a classroom using ten pictures, each of correct and incorrect sitting postures self-taken by a model [19]. After analyzing body landmarks using OpenPose, the accuracy of the final model exceeded 90% in detecting poor postures of the students. Piñero-Fuentes et al. used artificial intelligence (AI)-based posture estimations to determine participants’ inadequate postures when they were using a computer [20]. The training materials consisted of 151 video clips taken by 12 general subjects, from which 148 images were manually selected. They established a scoring scale to evaluate inadequate postures in these images. The performance of the final model in the four-item classification task achieved 84.1% accuracy. Nevertheless, differences in postures when using a computer between participants with and those without neck pain in real-world settings are still under-investigated. Han et al. developed a wearable monitoring system for forward head postures that utilizes a sensor-fusion device with a magnetometer pair and machine learning (ML) techniques [15].

In Chen’s study, “Sitting Posture Recognition Based on OpenPose” (2019) [19], 2D images were utilized to identify an individual’s sitting posture and assess its correctness. Similarly, Piñero-Fuentes’ research, “A Deep-Learning Based Posture Detection System for Preventing Telework-Related Musculoskeletal Disorders” (2021) [20], employed a 2D image analysis of joints to determine the sitting posture. It evaluates posture correctness by calculating the joint angles through specific formulas, aiming to prevent musculoskeletal disorders associated with telework. On the other hand, Han’s study, “Novel Wearable Monitoring System of Forward Head Posture Assisted by Magnet-Magnetometer Pair and Machine Learning” (2020) [15], focused on predicting neck postures using sensor data. This study introduced wearable technology specifically for measuring forward head posture (FHP), utilizing a three-axis magnetometer in combination with a small permanent magnet to aid in monitoring and predicting FHP through machine learning techniques.

The methodologies adopted in earlier studies involved the utilization of images and wearable sensing devices to capture spatial coordinates, movement directions, and kinematic information to analyze the subjects’ postures and movements. These methods often require advanced environmental setups and costly equipment. There is currently a lack of research that specifically focuses on non-specific neck pain. Therefore, here, we propose a method that utilizes videos captured by a basic 2D camera in conjunction with a machine-learning model. The research applied pose estimations based on machine learning to analyze images of subjects performing three separate computer-related tasks. Using a standard camera commonly found in typical work settings, our objective was to assess the posture of participants during real-world computer usage scenarios and create a classification model to determine if they are at risk of experiencing non-specific neck pain based on these observed traits. Additionally, the neck disability index (NDI) questionnaire [21], a tool frequently employed by clinical professionals, was utilized for clinicians to diagnose non-specific neck pain in subjects. NDI includes 10 sections that assess the level of pain and disability experienced by individuals with neck issues. These sections cover various aspects, including pain intensity, personal care, lifting, reading, headaches, concentration, work, driving, sleeping, and recreation. Each section is scored on a scale ranging from 0 to 5 or 0 to 4, depending on the questionnaire version, with higher scores indicating more severe pain or disability. This study aimed to enable the impartial assessment of neck pain patients’ postures by employing machine learning models. The utilization of such models is expected to enhance the assessments’ objectivity and consistency.

## 2. Materials and Methods

**Figure 1:**
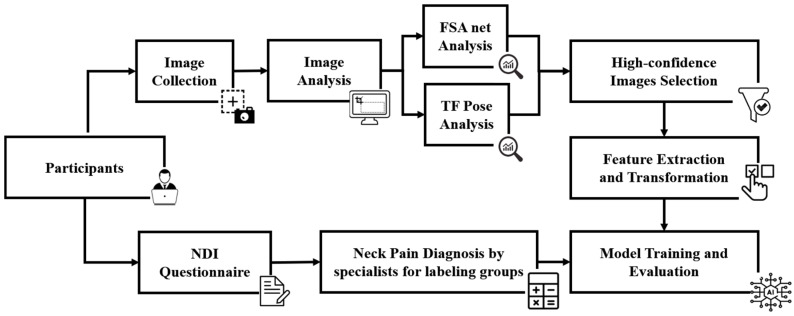
Workflow of the research.

The data used in this study were collected at Taipei Medical University (TMU) Hospital, and the project was approved by the TMU Institutional Review Board (Approval number: N202105005; Approval date: 27 May 2021). In total, 38 subjects (17 cases and 21 controls) were recruited from TMU Hospital. Patients with non-specific neck pain were evaluated by a physiatrist based on the NDI questionnaire and a physical examination to exclude any definite pathology. A 2D camera was used to capture images of participants completing three computer tasks over a period of thirty minutes. Pose estimation was performed using FSA Net and TF Pose. Predictive models were created using a variety of machine-learning approaches after selecting high-confidence images and performing feature extraction and transformation. The models were evaluated using nested cross-validation, and the hyperparameters were fine-tuned. Finally, an ensemble learning approach was employed to construct the final predictive model. The workflow is depicted in Figure 1.

### 2.1. Image Collection

We used a camera and a video capture application program to record images of subjects using a computer for 30 min. During this process, the subjects were required to perform three different tasks. First, a subject was required to type a sample text for the first 10 min. Second, the subject was required to play a video game only using the mouse, and the posture of the subject was recorded when using the mouse with one hand for 10 min. The final task was to watch a video for 10 min. We recorded the subject’s postures with their arms hanging naturally. We used a 2D camera (Logitech C920) to record videos, and the image resolution was up to 1920 × 1080. Back-end image analysis was performed, and a predictive model was built on a server with two Intel i9 10900K CPUs, an RTX 3090 GPU, and 128 G RAM. The three different tasks were designed to observe and record postural changes in the subjects when they used the computer with two hands, one hand, and no hands, respectively. Then, these images were used to analyze poses. In addition, the subjects were required to fill out a neck disability index (NDI) questionnaire. A physiatrist diagnosed patients with non-specific neck pain based on the questionnaire results, history taking, and clinical examination to evaluate the severity of neck pain.

### 2.2. Image Analysis

**Figure 2:**
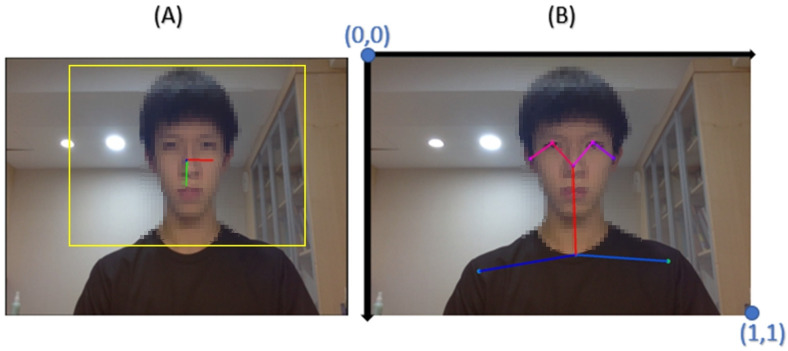
Image analysis results of (**A**) head poses, including angles of yaw, pitch, and roll and (**B**) positions of the eyes, nose, neck, and shoulders. The bounding box is displayed as a yellow rectangle. The top left point is the origin point (0,0).

The image analysis was composed of the fine-grained structure aggregation (FSA-Net) approach [22] and transformer-based pose estimation (TF pose) approach [23] using a Carnegie Mellon University (CMU) model. Fifteen raw features were collected from an image, including three types of head angles (yaw, pitch, and roll) (Figure 2A) and the x- and y-coordinates of the subject’s eyes, shoulders, nose, and neck in the image (Figure 2B).

Additionally, numerous features were also conducive to model training by feature transformation. In total, 1800 images were captured from the 30 min video recording of a subject, i.e., one frame per second. The subjects were required to sit upright and correct their position on the screen through a bounding box for the first 10 s (Figure 2A). The average values of these 10 s were used as a personal baseline, which was used to calibrate differences among subjects, such as body size or the relative position of the camera. During image selection, we removed images that were blurry or corrupted or could not be analyzed by checking whether they possessed estimated values of yaw, pitch, roll, and the coordinates of the eyes, shoulders, neck, and nose after image analysis. Additionally, the confidence scores generated by TF Pose were also used as a threshold for image selection. Images with a confidence score below Q1~1.5 of the interquartile range (IQR) were removed to reduce noise and false positives in the dataset.

**Figure 3:**
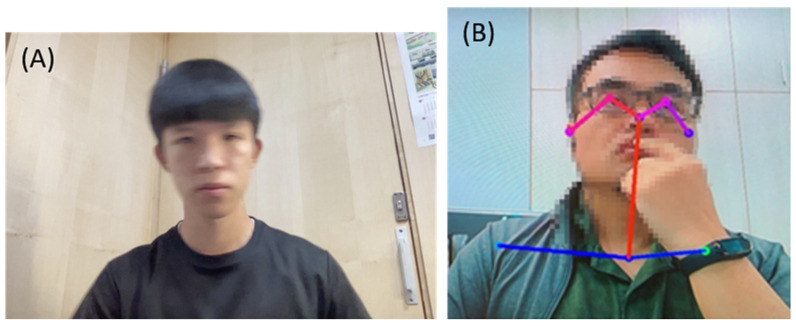
Example of images with missing values or low confidence: (**A**) The image is too blurry to analyze. (**B**) Threshold of left shoulder confidence score of this sample is 0.212. Left shoulder confidence score of this frame is 0.17, which is a low confidence score.

Some images were removed from the analysis due to missing values or low confidence after the pose estimation process. The statistical results showed that the average removal rate of images of subjects was about 9%. After reviewing the images, we found that the reasons for the missing information were mostly due to a subject’s rapid body movement or rapid head rotation during camera shooting (Figure 3A). Therefore, the captured image was too blurry to analyze. On the other hand, the reason for low confidence levels might have been that the user’s body points were blocked by other objects. For example, as shown in Figure 3B, a subject’s left wrist was mistakenly judged to be the left shoulder point. Therefore, through our image selection criteria, high-confidence images were collected to build a predictive model even though we did not employ a manual image-selection process.

### 2.3. Feature Extraction and Transformation

Pose and position information from images and the basic demographic information of the subject were converted into six feature types for the predictive model. These features were employed to delineate the profiles of the head and neck, encompassing various aspects such as neck angles, absolute and relative positions of the head and shoulders, identification of shoulder imbalances, and analysis of shoulder movements during computer usage; they are shown as follows:

1. Clinical data features: Five clinical features were obtained from the questionnaires: gender, age, height, body weight, and body mass index (BMI). They were used as training features for the predictive models.
2. Original pose features: There were 15 original pose features for each image, including 3 features of the head angles (yaw, pitch, and roll) and 12 coordinate features of each point in the image (position, *Po*), such as those for the eyes (Leye (x, y), Reye (x, y)), shoulders (Lshoulder (x, y), Rshoulder (x, y)), nose (Nose (x, y)), and neck (Neck (x, y)). The average value of each feature in a task was used as a representative feature for model training and prediction.
3. Offset pose features: As mentioned above, the average values of the original pose features in the first 10 s were used as a personal baseline, and offset pose features were calculated by subtracting the baseline value from the original pose features. Therefore, there were 15 offset pose features for each image.
4. Normalized position features: The coordinates generated from the TF pose were the absolute position, i.e., the upper left point of an image was set as the origin point (0,0). For each subject, we reset the origin point with its neck position to obtain a relative position. Therefore, 20 normalized position features of the eyes, nose, and shoulders were generated from the original pose and offset pose.
5. Status features: There were 25 status features generated from three parts. (1) To observe the status of the head pose, we transformed the angle value of each head pose (yaw, pitch, and roll) in an image into seven intervals based on its overall distribution in a task segmented by one, two, and three standard deviations (SDs). The average of the angle value in the baseline was regarded as μ. The proportions that fell within the intervals of a subject in a task were used as training features for the predictive models. For head postures (roll, jaw, pitch), we segmented their angles into seven intervals derived from each participant’s mean (µ) and standard deviation (SD) during tasks. Subsequently, we calculated the distribution ratios for each participant within these seven intervals to delineate and characterize their posture features. Seven intervals were introduced as Normal: µ − SD < angle < µ + SD; Mild_Right: µ − 2SD < angle < µ − SD; Moderate_Right: µ − 3SD < angle < µ − 2SD; Severe_Right: angle < µ − 3SD; Mild_Left: µ + SD < angle < µ + 2SD; Moderate_Left: µ + 2SD < angle < µ + 3SD; and Severe_Left: µ + 3SD <angle. (2) Additionally, we segmented the shoulder position into three intervals using a similar measure with a cutoff value of two standard deviations. To observe the status of shoulder imbalances, we transformed differences between the y-coordinate of the left and right shoulders in an image into three intervals based on the overall distribution of differences in a task segmented by two SDs. The average value of the shoulder difference of the baseline was regarded as μ. The proportions that fell within the intervals of a subject in a task were used as training features for the predictive models. (3) To observe the status of shrugging, we calculated the distribution of differences in the y-coordinate of a shoulder by subtracting its average value of the baseline in each image in a task, and one SD of the distribution was set as the threshold for detecting shrugging. A subject was detected as shrugging when both shoulders were simultaneously higher than a standard deviation. Thus, the shrugging proportions of a subject in a task were used as training features for the predictive models.
6. Variation features: There were 19 variation features generated from two parts: (1) By calculating the SD of 15 pose features, including head angles and the coordinates of each point, we recorded a subject’s body movements during different tasks. The average values of the SD in an image were used as training features for the predictive models. (2) The height difference between the left and right shoulders was observed when the subject was performing different tasks. Four features (Shoulder_Diff, Offset_Shoulder_Diff, ABS_Shoulder_Diff, ABS_Offset_Shoulder_Diff) were calculated using differences between the y-coordinate of the left shoulder and that of the right shoulder in an image with/without calibration by the baseline and taking the absolute value. The formulas are as follows:

$$\mathrm{Shoulder}\_\mathrm{Diff}=\mathrm{AVG}(\overset{1800}{\underset{i=1}{\sum }}\;\left(L\_\mathrm{Height}\;-\;R\_\mathrm{Height}\right))$$

$$\mathrm{Offset}\_\mathrm{Shoulder}\_\mathrm{Diff}=\mathrm{AVG}(\overset{1800}{\underset{i=1}{\sum }}\;\left(\left(L\_\mathrm{Height}\;-\;L\_\mathrm{baseline}\_\mathrm{height}\right)-\left(R\_\mathrm{Height}\;-\;R\_\mathrm{baseline}\_\mathrm{height}\right)\right))$$

$$\mathrm{ABS}\_\mathrm{Shoulder}\_\mathrm{Diff}=\mathrm{AVG}(\overset{1800}{\underset{i=1}{\sum }}\;\left(\left|L\_\mathrm{Height}\;-\;R\_\mathrm{Height}\right|\right))$$

$$\mathrm{ABS}\_\mathrm{Offset}\_\mathrm{Shoulder}\_\mathrm{Diff}=\mathrm{AVG}(\overset{1800}{\underset{i=1}{\sum }}\;\left(\left|\left(L\_\mathrm{Height}\;-\;L\_\mathrm{baseline}\_\mathrm{height}\right)-\left(R\_\mathrm{Height}\;-\;R\_\mathrm{baseline}\_\mathrm{height}\right)\right|\right))$$

After feature extraction and transformation, 94 pose-related features were generated from images in a task, including 15 original pose features, 15 offset pose features, 25 status features, 20 normalized position features, and 19 variation features. By calculating pose-related features of images from the typing task (10 min), gaming task (10 min), video-watching task (10 min), and the entire process (30 min), in addition to 5 clinical data features, there were 381 features per sample.

### 2.4. Model Training and Evaluation

**Figure 4:**
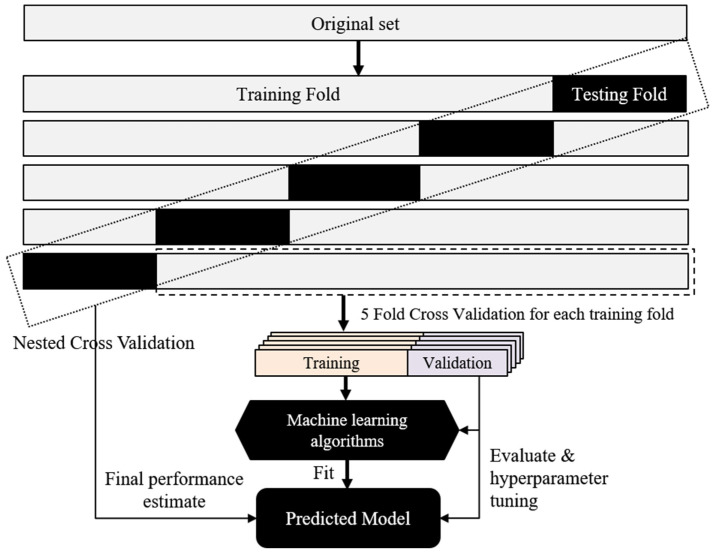
Model training and evaluation.

Random forest, support vector machine (SVM), extreme gradient boosting (XG Boost), and various machine learning methods were used to build a model for neck pain predictions. The Mann–Whitney U-test and Lasso (least absolute shrinkage and selection operator) methods were applied for feature selection. Additionally, bagging, AdaBoost, and gradient-boosting classifiers of ensemble learning were also adopted to construct the model. Regarding model evaluation, the performance of a model was mainly evaluated by its accuracy and area under the receiver operating characteristic curve (AUROC), and we also focused on the performance of other indicators such as recall, precision, specificity, and the F1-score. In order to improve the reliability of the model, we used nested cross-validation with stratified sampling to ensure that every sample was used as test data (Figure 4). The dataset was partitioned, allocating 80% for training and 20% for testing purposes. The training fold underwent assessment through a 5-fold cross-validation process. The parameters of the predictive model were fine-tuned with GridSearchCV. We then used the testing fold for model performance testing. Finally, the average of the results of five different testing folds was regarded as the performance of the nested cross-validation. The model parameters were as follows: random forest (n_estimators = 100, max_features = log2, and the others used the default setup), bagging (n_estimators = 50, bootstrap = True, bootstrap_features = True, max_features = 0.5, max_samples = 0.5, and the others used the default setup). In this study, the results of the repeated nested cross-validation were averaged as the final evaluation of model performance.

### 2.5. Testing Environment

The computer was placed 10 cm from the edge of the table on the user’s side, and the webcam tripod had a height of 24 cm. The height of the table and chair were 70 cm and 40 cm, respectively. The distance from the computer to the webcam tripod was 26 cm. Irrelevant individuals or items in the background that could lead to errors in image analysis were avoided. Tensorflow version 2.0.0 and Sklearn version 0.24.2 were used. For the front-end environment, we used a computer that could handle the paperwork. We used external cameras (Logitech C920) to record videos, and the image resolution was set to be up to 1920 × 1080. Compared to the default camera of the computer, the external camera had a wider shooting angle so that the image received was more complete.

## 3. Results

In total, 38 subjects participated in this study, including 15 males and 23 females. Most of the subjects had the habit of using computers in their normal daily life, whether working or in their free time. The study was conducted by recording images of these subjects using a computer as the target of analysis. The demographics of the subjects are shown in Table 1.

In total, 381 features were generated after feature extraction and transformation. In order to determine the correlations between features and targets, two methods were applied. The Mann–Whitney U-test was used to calculate *p* values of features between subjects with neck pain (case group) and those without it (control group). The features with a *p* value of <0.01 were selected, and 67 features in this study were chosen for further comparative analyses. Additionally, the Lasso method was also used for feature selection, which gives a weight to each feature during the training process, and then we selected features using those weights. In total, nine features were selected by the Lasso method. There were 8 intersection features (Table 2) and 68 union features (Table S1) between the two methods. We regarded the 68 union features as training features for the prediction model to compare the predictive performances between all features and selected features.

After feature selection, all features (381 features) and selected features (68 features) were used to construct the predictive model using various machine learning methods, including random forest, XGBoost, and logistic regression. Table 3 shows the predictive performances of the models with different features and machine learning methods. The results showed that the random forest had the best performance, whether using all features or selected features. The predictive performance of the model was significantly improved using the selected features. The accuracy reached 81.4%, the F1-score reached 77.4%, and the AUROC reached 81.2%.

In order to improve the performance, GridSearchCV was applied to fine-tune the hyperparameters of the model. The average performances of the five-fold nested cross-validation with five repetitions after fine-tuning with GridSearchCV (n_estimator = 100, max_features = log2) are shown in Table 4. The overall model performance had improved compared to the previous performance (Table 3). The average accuracy reached 83%, the F1-score reached 80%, and the AUROC reached 83%.

**Figure 5:**
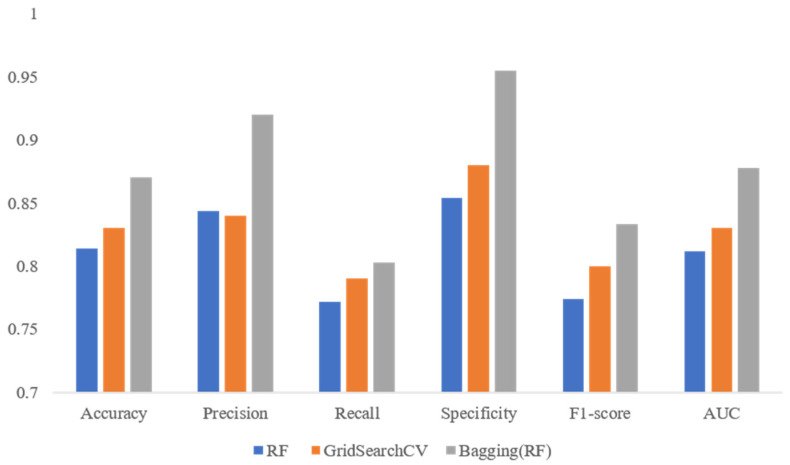
Changes in predictive performance of the model (random forest (RF)) after fine-tuning of hyperparameters (GridSearchCV) and ensemble learning (bagging).

Bagging, AdaBoost, and gradient-boosting classifiers of ensemble learning were applied to enhance the predictive performance of the model. Here, we used the random forest algorithm as a weak classifier. The performances of the three methods are shown in Table 5. The results showed that bagging had the best predictive performance. The accuracy reached 87%, the F1-score reached 83.3%, the AUROC reached 87.8%, and the other indicators were all >0.8. The changes in the performance of the predictive models after fine-tuning the hyperparameters and ensemble learning are shown in Figure 5. The results showed that the performances of various indicators of the predictive model improved with step-by-step improvements in the training data and machine learning methods.

Compared to other related studies, there is currently a limited body of research specifically addressing non-specific neck pain. Piñero-Fuentes et al. [20] developed a posture detection system utilizing deep learning to assess participants’ postures, specifically the back, shoulders, and neck, which fell into one of four ranges (0, 1, 2, or 3). These ranges are then compared to the associated risk of developing musculoskeletal diseases. The system’s accuracy for predicting the four range classes and two range classes (0–1 or 2–3) was 0.81 and 0.93, respectively.

**Figure 6:**
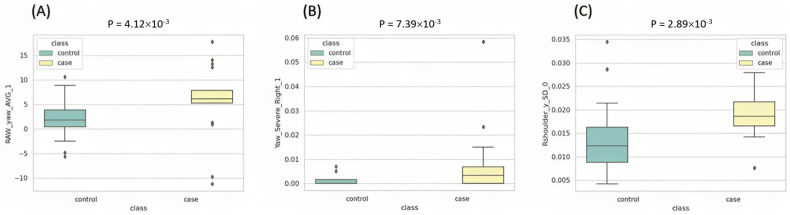
Boxplot of (**A**) RAW_yaw_AVG_1, (**B**) Yaw_Severe_Right_1, and (**C**) Rshoulder_y_SD_0 in the case and control groups. Abbreviations: RAW_yaw_AVG_1: the average value of yaw during the typing task (0~10 min); Yaw_Severe_Right_1: the proportion of yaw of the entire data that fell in the category of severe during the typing task (0~10 min); Rshoulder_y_SD_0: the standard deviation of the y-coordinate of the right shoulder during the entire process (0~30 min).

There were 19, 16, and 13 features selected from the typing task, gaming task, and video-watching task, respectively (Table S1). Typing and using a computer with both hands on the keyboard is the most common posture for general computer users, such as administrative clerks, programmers, etc. The analysis results showed that the typing task generated more discriminative features than the gaming task, where a mouse is used with one hand, and the video-watching task, where no hands are used. For example, as shown in Figure 6A, RAW_yaw_AVG_1, which represents the average value of the yaw of the subject during the typing task, was observed with values closer to 0 in control samples compared to case samples. This means that subjects in the control group had smaller head deflection angles during the typing task. As shown in Figure 6B, Yaw_Severe_Right_1, which represents the proportion of time that a subject’s head was severely turned to the right during the typing task, was observed at significantly higher proportions in case samples than control samples. These discriminative features could be helpful for constructing a highly accurate prediction model.

Additionally, variation features were the most frequently observed (25 features) in Table S1 compared to other types of features, such as pose, position, and status features. This means that changes in the movement of each subject’s eyes, shoulders, nose, and neck during the tasks could be very useful and important for training the model. For example, as shown in Figure 6C, Rshoulder_y_SD_0, which represents the SD of the y-coordinate value of the right shoulder of the subject during the entire process (30 min), was observed to have lower values in control samples than in case samples.

**Figure 7:**
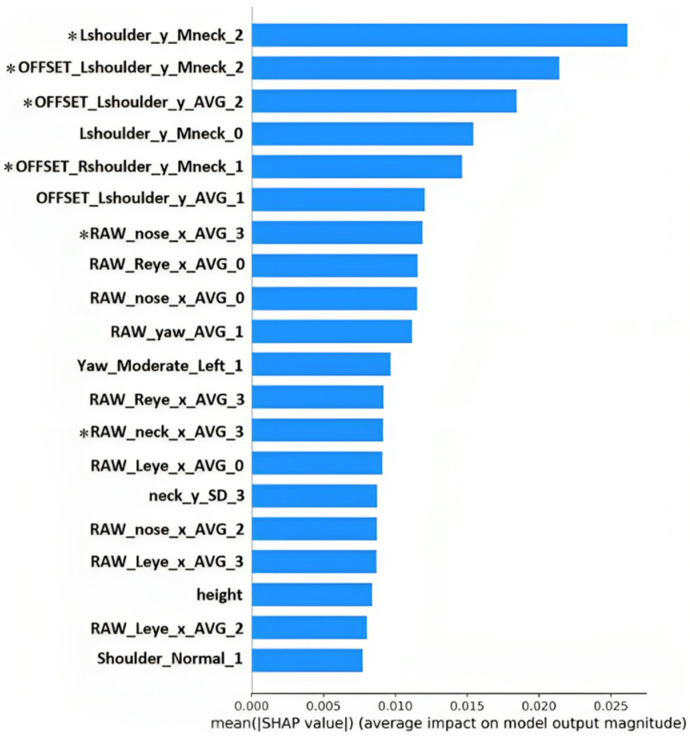
SHAP importance plot of the top 20 features. *, intersection features selected by Lasso and the U-test. Abbreviations of features: RAW, raw features generated from the TF Pose or FSA-Net; Offset, offset features generated by subtracting the subject’s baseline value; Mneck, normalized features generated by resetting the origin point (0,0) with the subject’s neck position; [0/1/2/3], features generated from the whole/typing/gaming/video-watching task; [R/L][shoulder/eye], right/left shoulder/eye; [eye/nose/neck/shoulder]_[x/y], average values of the x/y-coordinates of images from eye/nose/neck/shoulder; Shoulder_[Normal/Imbalance], status feature of the shoulders; Shoulder_Diff, difference between the height (y-coordinate) of two shoulders; [Yaw/Pitch/Roll]_AVG, average value of angles of images of yaw, pitch, or roll; [Yaw/Pitch/Roll]_[Normal/Mild/Moderate/Severe]_[Right/Left], head pose features generated from the proportions of angle values located in seven intervals based on the subject’s overall distribution in a task segmented by one, two, or three standard deviations and annotated with head direction (right/left); ABS, the absolute value of a feature; SD, the standard deviation of a feature.

Furthermore, we scored all features by applying the SHAP (SHapley Additive exPlanations) method and listed the top 20 features in Figure 7. The results showed that six of the eight intersection features (Table 2) between the Mann–Whitney U-test and Lasso also appeared in the top 20, which showed that the selected features were significant and useful for model construction.

## 4. Discussion

This is the first predictive model for accessing non-specific neck pain through pose estimation from images. This study evaluated three frequently executed computer tasks; various features were extracted and transformed for an ensemble learning model through the pose estimation of images captured by a 2D camera. Through the feature selection using U-tests and Lasso, 19, 16, and 13 features were selected from the typing task, gaming task, and video-watching task, respectively. This shows that there are different factors affecting neck pain in different situations of computer use that should be noted. Our final model achieved an accuracy, recall, precision, specificity, F1-score, and AUROC of above 0.8 for all.

Prolonged use of mobile phones, computers, and other electronic products with an incorrect posture is associated with neck pain [24]. Mao et al. conducted a study of pose estimations based on the framework of OpenPose and DeepPose [23]. A transformer encoder and decoder were added to the model to improve model performance. The authors used portrait pictures from the COCO and MPII datasets to train the model. The output included 18 body landmarks such as the eyes, nose, neck, shoulders, and elbows. The accuracy of the final model was 90.4%. Yang et al. published a study with AFLW2000 and BIWI as the training and test datasets [22]. By inputting facial pictures at different orientations and angles for training, the values of the three axes of each picture (yaw, pitch, and roll) were output. The research results and ground truth provided by the dataset were used to calculate the mean absolute error (MAE), and the best prediction performance of the model was MAE = 3.60 (BIWI dataset). Cao et al. [25] published a model using the COCO and MPII datasets in 2019. The training model trained and predicted 18 points defined by the portrait pictures in the dataset. The output of each point contained the x- and y-coordinates and the confidence level value of the judgment coordinates. The prediction performance of the final convolutional neural network (CNN) model had an average precision of 0.653 (COCO). In 2014, Toshev et al. published a study on data training and prediction using the Frames Labeled In Cinema (FLIC) and Leeds Sports Pose (LSP) datasets [26], which were used to train the model to predict the points of the upper limbs and the whole body, respectively. The predictions of the final deep neural network (DNN) based on a regressor model for the two were percentages of correct parts (PCPs) of 0.61 (FLIC) and 0.69 (ImageParse dataset).

We recognized several specific postures in the subjects with and without non-specific neck pain. These features may reflect the asymmetry of the neck and shoulder positions, and an abnormal head position is characteristic of patients with non-specific neck pain. Our findings can be used to develop a real-time feedback tool when the individual uses a laptop to be alerted when they adopt these poor postures. In addition, the machine learning algorithm may also help in performing large-scale posture screening in the classroom or office for public health. Finally, our tool can monitor the posture changes after a specific training or health education program for non-specific neck pain subjects.

Our findings are generally consistent with previous studies regarding posture features in non-specific neck pain. Some abnormal postures have been recognized in subjects using computers, such as a forward head (holding the head in front of its natural position over the cervical spine) and rounded shoulders (moving the shoulder forward from its natural position) postures. A forward head causes a decreased angle of cervical flexion and an increased staring angle. It has been demonstrated that changes in the position of the head can increase the load put onto the musculoskeletal system [27]. It also has been found that muscle overactivity in the sternocleidomastoid, upper trapezius, cervical vertebral spinae, and thoracic vertebrae spinae muscles occurs in the forward head posture [28]. Trapezius and sternocleidomastoid muscle overactivity can be noted by unconsciously shrugging shoulders. Increased biomechanical loading and muscle overactivity can make these structures prone to tissue injuries and degeneration. In addition, posture asymmetry while using the computer has been reported to be linked with lower health-related quality of life in some physical domains, such as pain, in adolescents [29].

As a cross-sectional observational study, the cause-and-effect relationship between non-specific neck pain and poor posture cannot be determined in the present study. The association between non-specific neck pain and poor posture could be bi-directional. Past research showed increased activity of neck–shoulder muscles, such as the upper trapezius and cervical erector spinae, during typing tasks in chronic neck pain patients under different physical conditions [30]. The hyperactivity of these stabilizing muscles may further cause the shortening of muscles and exacerbate posture. Furthermore, a systematic review found that age is an important confounding factor in the relationship between poor posture and neck pain. The adults with neck pain showed an increased frequency of the forward neck posture compared to asymptomatic adults [31].

As shown in Table 4, nested cross-validation was independently performed five times to increase the reliability of the model and prevent overfitting. The model’s accuracy ranged from 0.79 to 0.86, and the precision varied between 0.75 and 0.92. The AUROC results from the five tests ranged from 0.8 to 0.88, suggesting some level of stability in our proposed model. Since the sample size of the model needs to be larger to ensure both generalizability and clinical applicability, we plan to increase the cohort size in the future to create a more comprehensive and easily adaptable model. Additionally, prolonged and consistent observation and assessment of posture while using a computer will be taken into account. As shown in Table 5, although the final model achieved an accuracy of 0.824 and an AUROC of 0.874, there are noticeable differences between sensitivity and specificity, with values of 0.803 and 0.955, respectively, indicating biased classification. This may be because the sample size of the non-specific neck pain group was smaller than the control group. It also highlights the necessity of discovering more representative characteristics to distinguish between the two groups. Therefore, to reduce biased classification, additional samples should be gathered not only to attain balance in sample size between the two groups but also to ensure that demographic characteristics are matched in future studies. Additionally, more potential representative characteristics, such as side-view photography, could be taken into consideration.

A limited sample size of the participants was considered in the current study. The model needs a larger number of samples and a more balanced dataset to be validated. In addition, the current study utilized a cross-sectional design. Long-term variations in posture during computer use could significantly contribute to the onset of neck pain. The present study used simulated conditions to record the participants’ postures using computers. In real-world settings, the setup when using computers, including screen height and distance, can be more complex. These may also affect the generalization of the model. This study only used 30 min of recording to analyze posture. It is possible that posture could change after longer periods of computer use due to fatigue. Finally, the model we developed in the present study should be verified by external validation data to examine its generalizability.

## 5. Conclusions

The research contribution of this study is to provide a convenient and cost-effective means of assessment using a basic 2D camera without the need for complicated environmental setups. Utilizing pose estimation, feature transformations, and parameter optimization, our predictive model employed an ensemble learning approach to achieve a good performance, with an 87% accuracy, 92% precision, 80.3% recall, 95.5% specificity, and an AUROC of 0.878. The results of this study provide a pragmatic method for evaluating suboptimal postures in real-life computer usage scenarios. This method has the potential to aid clinicians in assessing postures linked to neck pain. In addition, our model can enhance self-awareness regarding incorrect postures among computer users, which, in turn, may help them develop proactive strategies for posture management.

## Figures and Tables

**Table 1:** Demographics of subjects.

Feature	Statistics	Controls (N = 21)	Cases (N = 17)
Gender (female)	n (%)	10 (47.6)	13 (76.5)
Age (years)	Mean (SD)	31.5 (6.90)	35.8 (16.48)
Height (cm)	Mean (SD)	168.2 (6.75)	160.9 (6.84)
Weight (kg)	Mean (SD)	73.5 (19.68)	62.5(12.17)
BMI (kg/m^2^)	Mean (SD)	25.9 (6.02)	24.1 (4.58)
NDI	Mean (SD)	NA	3.2 (1.9)

SD, standard deviation; BMI, body mass index; NA, not applicable.

**Table 2:** Intersection features between the U-test and Lasso method.

Feature	Cases Mean Value	Controls Mean Value	*p* Value (U-Test)
RAW_Rshoulder_x_AVG_1	0.286	0.326	0.001
OFFSET_Lshoulder_y_AVG_2	−0.044	−0.075	<0.001
RAW_nose_x_AVG_3	0.475	0.516	<0.001
RAW_neck_x_AVG_3	0.493	0.527	<0.001
Rshoulder_y_SD_0	0.019	0.014	0.003
OFFSET_Rshoulder_y_Mneck_1	−0.009	0.002	0.001
Lshoulder_y_Mneck_2	0.006	−0.017	<0.001
OFFSET_Lshoulder_y_Mneck_2	0.009	−0.007	<0.001

Abbreviations: RAW_Rshoulder_x_AVG_1: average value of the x-coordinate of the right shoulder during the typing task (0~10 min); OFFSET_Lshoulder_y_AVG_2: average value of the y-coordinate of the left shoulder after subtracting the baseline from RAW features during the gaming task (11~20 min); RAW_nose_x_AVG_3: average value of the x-coordinate of the nose during the video-watching task (21~30 min); RAW_neck_x_ AVG_3: average value of the x-coordinate of the neck during the video-watching task (21~30 min); Rshoulder_y_SD_0: standard deviation of the y-coordinate of the right shoulder during the entire process (0~30 min); OFFSET_Rshoulder_y_Mneck_1: normalized feature of the y-coordinate of the right shoulder after subtracting the baseline from RAW features during the typing task (0~10 min); Lshoulder_y_Mneck_2: normalized feature of the y-coordinate of the left shoulder during the gaming task (11~20 min); OFFSET_Lshoulder_y_Mneck_2: normalized feature of the y-coordinate of the right shoulder after subtracting the baseline from RAW features during the gaming task (11~20 min).

**Table 3:** Predictive performances of the models with different features and machine learning methods.

Feature	Total Features (N = 381)			Selected Features (N = 68)		
Machine Learning	Random Forest	XGBoost	Logistic Regression	Random Forest	XGBoost	Logistic Regression
Accuracy	**0.76**	0.69	0.584	**0.814**	0.748	0.742
Precision	**0.81**	0.718	0.604	**0.844**	0.738	0.786
Recall	**0.642**	0.588	0.518	**0.772**	0.666	0.69
Specificity	**0.876**	0.784	0.708	**0.854**	0.8	0.842
F1-score	**0.668**	0.606	0.482	**0.774**	0.668	0.67
AUROC	**0.76**	0.686	0.612	**0.812**	0.734	0.764

AUROC, area under the receiver operating characteristic curve. The highest values among different ML approaches are shown in bold.

**Table 4:** The average performance of the random forest after fine-tuning.

GridSearchCV	Test 1	Test 2	Test 3	Test 4	Test 5	Avg.
Accuracy	0.86	0.79	0.81	0.84	0.84	0.83
Precision	0.92	0.85	0.75	0.85	0.78	0.84
Recall	0.84	0.75	0.72	0.85	0.87	0.79
Specificity	0.87	0.87	0.88	0.88	0.89	0.88
F1-score	0.87	0.77	0.75	0.81	0.8	0.8
AUROC	0.86	0.81	0.8	0.86	0.88	0.83

Avg., average; AUROC, area under the receiver operating characteristic curve.

**Table 5:** Performances of the ensemble learning predictive models.

	RF Model	Bagging	AdaBoost	Gradient Boosting
Accuracy	0.83	**0.87**	0.824	0.794
Precision	0.84	**0.92**	0.85	0.84
Recall	0.79	**0.803**	0.794	0.732
Specificity	0.88	**0.955**	0.878	0.874
F1-score	0.8	**0.833**	0.786	0.748
AUROC	0.83	**0.878**	0.836	0.802

RF, random forest; AUROC, area under the receiver operating characteristic curve. The highest values among different ensemble learning approaches are shown in bold.

## Data Availability

Data is not shared due to privacy concerns regarding personal facial images.

## Supplementary Materials

The following supporting information can be downloaded at: https://www.mdpi.com/article/10.3390/life13122292/s1, Table S1: The features used for training.
