# Supplementary material for: Assessing Non-Specific Neck Pain through Pose Estimation from Images Based on Ensemble Learning
Source: Life (Basel). 2023 Nov 30;13(12):2292. doi: 10.3390/life13122292 (PMC10744896; doi:10.3390/life13122292)
Supplement: Supplementary file 1 [file life-13-02292-s001.zip › life-2700433-supplementary.pdf]

**Table S1.** The features used for training

| Features | Case Mean Value | Control Mean Value | P value (U Test) | Selected By Lasso |
|---|---|---|---|---|
| Offset_Lshoulder_y_Mneck_2 | 0.009 | -0.007 | <0.001 | Y |
| Lshoulder_y_Mneck_2 | 0.006 | -0.017 | <0.001 | Y |
| OFFSET_Lshoulder_y_AVG_2 | -0.044 | -0.075 | <0.001 | Y |
| RAW_neck_x_AVG_3 | 0.493 | 0.527 | <0.001 | Y |
| RAW_nose_x_AVG_3 | 0.475 | 0.516 | <0.001 | Y |
| Offset_Rshoulder_y_Mneck_1 | -0.009 | 0.002 | 0.001 | Y |
| RAW_Rshoulder_x_AVG_1 | 0.286 | 0.326 | 0.001 | Y |
| Rshoulder_y_SD_0 | 0.019 | 0.014 | 0.003 | Y |
| Yaw_Mild_Left_2 | 0.066 | 0.101 | >0.05 | Y |
| Lshoulder_y_Mneck_0 | 0.003 | -0.013 | <0.001 | |
| RAW_Reye_x_AVG_3 | 0.437 | 0.478 | <0.001 | |
| RAW_nose_x_AVG_0 | 0.482 | 0.518 | <0.001 | |
| Lshoulder_y_SD_1 | 0.014 | 0.008 | <0.001 | |
| RAW_Leye_x_AVG_3 | 0.525 | 0.561 | <0.001 | |
| RAW_Rshoulder_x_AVG_0 | 0.285 | 0.326 | 0.001 | |
| Offset_Shoulder_Diff_2 | 0.025 | 0.048 | 0.001 | |
| RAW_Reye_x_AVG_0 | 0.439 | 0.475 | 0.001 | |
| RAW_Reye_x_AVG_2 | 0.436 | 0.471 | 0.001 | |
| nose_y_SD_1 | 0.022 | 0.014 | 0.001 | |
| Leye_y_SD_1 | 0.021 | 0.013 | 0.001 | |
| RAW_Leye_x_AVG_0 | 0.532 | 0.563 | 0.001 | |
| height | 160.912 | 168.238 | 0.001 | |
| RAW_nose_x_AVG_2 | 0.476 | 0.513 | 0.001 | |
| Reye_y_SD_1 | 0.021 | 0.013 | 0.001 | |
| Rshoulder_y_SD_3 | 0.015 | 0.01 | 0.001 | |
| RAW_Reye_x_AVG_1 | 0.446 | 0.479 | 0.001 | |
| RAW_neck_x_AVG_0 | 0.499 | 0.528 | 0.002 | |
| Leye_y_SD_0 | 0.025 | 0.017 | 0.002 | |
| RAW_Leye_x_AVG_2 | 0.53 | 0.562 | 0.002 | |
| RAW_Rshoulder_x_AVG_3 | 0.288 | 0.333 | 0.002 | |
| nose_y_SD_3 | 0.022 | 0.013 | 0.002 | |
| Lshoulder_y_Mneck_3 | 0.001 | -0.011 | 0.002 | |
| RAW_Rshoulder_x_AVG_2 | 0.284 | 0.323 | 0.002 | |
| Offset_ABS_Shoulder_Diff_2 | 0.029 | 0.048 | 0.002 | |
| OFFSET_Lshoulder_y_AVG_0 | -0.049 | -0.074 | 0.002 | |
| OFFSET_Lshoulder_y_AVG_3 | -0.049 | -0.069 | 0.002 | |
| Leye_y_SD_3 | 0.019 | 0.011 | 0.002 | |
| Shoulder_Normal_1 | 0.568 | 0.269 | 0.002 | |
| RAW_neck_x_AVG_2 | 0.5 | 0.529 | 0.002 | |
| Offset_Lshoulder_y_Mneck_0 | 0.007 | -0.003 | 0.002 | |
| Shoulder_Imbalance_Left_2 | 0.097 | 0.41 | 0.003 | |
| RAW_Lshoulder_x_AVG_3 | 0.68 | 0.713 | 0.003 | |
| Offset_Shoulder_Diff_0 | 0.029 | 0.047 | 0.003 | |
| Yaw_Moderate_Left_1 | 0.017 | 0.052 | 0.003 | |
| Shoulder_Normal_0 | 0.689 | 0.412 | 0.003 | |
| nose_y_SD_0 | 0.027 | 0.019 | 0.003 | |

| | | | |
|---|---|---|---|
| Reye_y_SD_0 | 0.025 | 0.017 | 0.003 |
| Offset_ABS_Shoulder_Diff_0 | 0.032 | 0.048 | 0.004 |
| Reye_y_SD_3 | 0.019 | 0.012 | 0.004 |
| RAW_yaw_AVG_1 | 5.665 | 1.955 | 0.004 |
| OFFSET_Lshoulder_y_AVG_1 | -0.054 | -0.078 | 0.004 |
| Shoulder_Diff_0 | -0.011 | 0.004 | 0.004 |
| neck_y_SD_3 | 0.012 | 0.008 | 0.004 |
| RAW_nose_x_AVG_1 | 0.49 | 0.522 | 0.004 |
| Shoulder_Diff_2 | -0.015 | 0.005 | 0.004 |
| Lshoulder_y_SD_0 | 0.019 | 0.014 | 0.005 |
| Offset_ABS_Shoulder_Diff_1 | 0.029 | 0.044 | 0.005 |
| Leye_y_SD_2 | 0.02 | 0.013 | 0.005 |
| RAW_neck_x_AVG_1 | 0.506 | 0.53 | 0.005 |
| Offset_Shoulder_Diff_1 | 0.026 | 0.042 | 0.006 |
| RAW_Lshoulder_x_AVG_2 | 0.697 | 0.723 | 0.007 |
| Yaw_Severe_Right_1 | 0.008 | 0.001 | 0.007 |
| Lshoulder_y_Mneck_1 | 0.002 | -0.01 | 0.007 |
| RAW_yaw_AVG_0 | 6.367 | 2.98 | 0.008 |
| RAW_Lshoulder_x_AVG_0 | 0.694 | 0.719 | 0.008 |
| OFFSET_neck_y_AVG_1 | -0.038 | -0.054 | 0.008 |
| neck_y_SD_1 | 0.011 | 0.007 | 0.008 |
| nose_y_SD_2 | 0.022 | 0.015 | 0.009 |

Abbreviation of components of features: RAW: raw feature generated from TF Pose or FSA net; Offset: offeset features generated by raw feature subtracting the subject's baseline value; _Mneck: normalized features generated by reset the original point (0,0) with the subject's neck position; _[0/1/2/3]: features generated from whole/typing/gaming/video-watching tasks, respectively; [R/L][shoulder/eye]: Righr/Left Shoulder/Eye; [eye/nose/neck/shoulder]_[x/y]: the average value of x/y-coordinate of images from eye/nose/neck/shoulder; Shoulder_[Normal/Imbalance]: status feature of shoulders; Shoulder_Diff: the difference between the height (y-coordinate) of two shoulders; [Yaw/Pitch/Roll]_AVG: the average value of angels of images from yaw, pitch, and roll; [Yaw/Pitch/Roll]_[Normal/Mild/Moderate/Severe]_[Right/Left]: head pose features generated from the proportions of angle values located in 7 intervals based on the subject's overall distribution in a task segmented by one, two and three standard deviations and annotated with head direction (right/left); ABS: the absolute value of the feature; SD: the standard deviation of the features.
